# IgA and IgG against *Mycobacterium tuberculosis* Rv2031 discriminate between pulmonary tuberculosis patients, *Mycobacterium tuberculosis*-infected and non-infected individuals

**DOI:** 10.1371/journal.pone.0190989

**Published:** 2018-01-26

**Authors:** Fekadu Abebe, Mulugeta Belay, Mengistu Legesse, Franken K. L. M. C., Tom H. M. Ottenhoff

**Affiliations:** 1 University of Oslo, Faculty of Medicine, Institute of Health and Society, Department of Community Medicine and Global health, Oslo, Norway; 2 Center for Immuno-biology, Bart’s and The London School of Medicine and Dentistry, Queen Mary University of London, London, United Kingdom; 3 Addis Ababa University, Aklilu Lemma Institute of Pathobiology, Addis Ababa, Ethiopia; 4 Department of Infectious Diseases, Leiden Medical Center, Leiden, the Netherlands; Institut de Pharmacologie et de Biologie Structurale, FRANCE

## Abstract

As part of a major project to investigate protective and diagnostic immune markers against tuberculosis (TB), we measured antibody isotype responses to *Mycobacterium tuberculosis* (*Mtb*) antigens (LAM, Rv2031, and HBHA) in cohorts of 149 pulmonary tuberculosis patients (PTBP), 148 household contacts (HHCs), and 68 community controls (CCs) in an endemic setting. ELISA was used to measure levels of IgA, IgG, and IgM from sera of cohorts at baseline, and at 6 and 12 months from entry. The results show that there were significant differences in IgA, IgG, and IgM responses to the different antigens and in the three cohorts. At baseline, the level of IgM against RV2031 and LAM did not vary between cohorts, but the levels of IgA and IgG against Rv2031 were significantly higher in PTB patients than HHCs and CCs, followed by HHCs, and the lowest in CCs. In patients, there was a significant variation in antibody responses before and after chemotherapy. The levels of IgA and IgG against HBHA, and IgA against Rv2031 decreased significantly and remained low, while IgA and IgG against LAM increased significantly and remained high following chemotherapy. However, the levels of IgM against Rv2031 and LAM increased at 6 months but decreased again at 12 months. IgM against HBHA did not show any significant variation before and after chemotherapy. Similarly, there were also significant variations in antibody responses in HHCs over time. Our results show that there are significant variations in IgA, IgG and IgM responses to the different antigens and in the three cohorts, implying that not all antibody isotype responses are markers of clinical TB. In addition, the current and previous studies consistently show that IgA and IgG against Rv2031 discriminate between clinical disease, *Mtb*-infected and non-infected individuals.

## Introduction

Tuberculosis (TB) caused mainly by *Mtb* remains one of the leading cause of death due to an infectious agent. According to the World Health Organization [1], there were 1.8 million deaths and 10.4 million clinical TB patients globally in 2015. In addition, it is believed that an estimated one-third of the global population is *Mtb* infected [1]. The only licensed TB vaccine currently in use, BCG does not control transmission. Efforts to replace BCG with an efficacious vaccine or to augment very little, partly because of lack of knowledge about correlates of protective immunity [2–5]. Efforts made to develop an efficacious vaccine based on cell-mediated immunity (mainly interferon-gamma production by CD4+ T cells) did not yield the desired results. In recent years, however, several studies from animal models and epidemiological studies have shown that antibody isotypes (especially IgA) are protective against TB [6–11]. *Mycobacterium tuberculosis* employs different virulent factors (antigens) for entry, invasion, and persistence and multiplication within the host cell. Lipo-arabinomannan (LAM), which is one of the major components of *Mtb* cell wall is associated with virulence and immuno-pathology, including inhibition of interferon-gamma-mediated macrophage activation [12], inhibition of T cell proliferation [13] or induction of T cell anergy [14, 15], inhibition of IL-12 production [16], inhibition of neutrophil recruitment [17], and inhibition of dendritic cell function and *Mtb*-induced apoptosis [18]. LAM is also involved in inhibition of Kinase C activities and in scavenging cytotoxic oxygen free radicals [19], and phagosomal maturation [20]. Moreover, TB associated clinical manifestations, namely fever, weight loss, and tissue necrosis have been attributed to LAM-induced cytokine production [21].

Similarly, Rv2031, the 16-kDa heat shock protein (hspX) of *Mtb* (also known as alpha crystalline) is another virulence factor involved in persistence of the bacilli in the host cell during latency. It is also an immuno-dominant protein predominantly produced during the stationary phase and is believed to play a critical role in maintaining long-term protein stability and long term survival of the pathogen [22, 23].

The 28-kDa, heparin-binding hemagglutinin (HBHA) of *Mtb* is a surface protein which has been shown to promote extra-pulmonary dissemination of *Mtb* by facilitating *Mtb*-epithelial cell attachment [24]. As one of *Mtb* virulence factors, it is known for inhibiting autophagy through induction of cytoplasmic reticulum stress-mediated apoptosis through generation of reactive oxygen intermediates and cytosolic ca^2+^ in murine macrophages [25]. While detection of LAM in urine is currently used in the diagnosis of TB, especially in TB/HIV co-infected individuals [26], the potential of Rv2031 [27] and HBHA for diagnosis [28, 29] and as candidate vaccine [30, 31] is being investigated.

The current study is part of a major project to assess protective and diagnostic immune markers using immuno-dominant antigens of *Mtb* in the population in a setting of high endemicity. Earlier, we have reported cytokine responses against immuno-dominant antigens such as recombinant early secreted antigen-6 and culture filtrate protein-10 (ESAT-/CFP-10) [32], HBHA [9], LAM [33] and Tv2031 [34]. In this paper, we present IgA, IgG, and IgM responses to LAM, RV2031, and HBHA in cohorts of pulmonary TB patients (PTBP), their household contacts (HHCs), and community controls (CCs).

## Materials and methods

### Study setting

The study was conducted in an endemic setting in Addis Ababa, Ethiopia, with a population of 2.6 million. Out of 24 health centers that provide services to directly observed treatment short course (DOTS), 7 were selected for the current study. Smear positive PTBP were recruited before initiation of treatment. Household contacts (HHCs) living in the same house with smear positive PTBP were screened for TB using clinical assessment and chest x-ray, and followed up for 12 months. AFB and culture were done for those with productive cough.

### Participants and data collection

Participants were recruited as described previously [35]. Briefly, smear positive PTBP were recruited prospectively before the initiation of anti-TB treatment. At the same time, HHCs, living with smear positive PTBP and healthy community controls (CCs) with no history of TB or known exposure to PTBP were recruited. Contacts had no evidence of active TB.

Clinical assessment including weight, height and BCG scar examination was done for all participants. QuantiFERON-TB Gold In-Tube test was used to screen HHCs, and CCs for *Mtb* infection as described earlier [35]. Chest x-ray, smear microscopy and sputum culture were used to rule out TB in HHCs and CCs. Patients were treated with anti-TB drugs for 6 months; however, HHCs were not given prophylactic treatment in line with the national guideline [36]. Screening for HIV infection was done according to the national guideline [37], and only those without HIV infection were included in the study. Participants were between 18 and 60 years of age with no apparent immunosuppressive conditions. Patients and HHCs were followed up for 12 months with clinical examination and sample collection at entry, 6 and 12 months. For CCs, blood samples were collected at entry.

### Antibody ELISA

Serum levels of antibody isotypes, IgA, IgG, and IgM against LAM, Rv2031, and HBHA were measured using ELISA as described earlier [9]: Nunc MaxiSorp ELISA plates (Sigma Aldrich, Germany) were coated with LAM (10μg/ml); Rv2031 (10μg/ml); and HBHA (4μg/ml) diluted in carbonate-bicarbonate coating buffer (Sigma-Aldrich, Germany) and incubated overnight at 4°C. Plates were washed with PBS containing 0.05% Tween 20 and blocked with PBS containing 2% BSA (Sigma-Aldrich, Germany) overnight at 4°C. After washing, 100μl of samples diluted 1:100 (IgG) and 1:50 (IgM, IgA) in PBS containing 1% BSA and 0.05% Tween 20 were added each well and plates incubated at room temperature (RT) for 2 hour. After washing, 100μl of goat anti-human IgG, IgA, and IgM antibodies (biotinylated, 0.5 μg/ml) (Mabtech, Sweden) diluted at 1:1000 in PBS containing 1% BSA and 0.05% Tween 20 were added into each well of the respective plates. Plates were incubated at RT for an hour and after washing, 100μl streptavidin-horse radish peroxidase enzyme (Mabtech, Sweden) diluted at 1:2000 in PBS containing 1% BSA and 0.05% Tween 20 was added into each well. After washing 100μl of 3, 3`5, 5`-tetramethylbenzidine substrate tablets (Sigma-Aldrich, Germany) diluted in phosphate citrate buffer with sodium perborate (Sigma-Aldrich, Germany) was added into each well. After 15 minutes of incubation, reaction was stopped with 2N sulfuric acid and read at 450nm. Optical density (OD) values were used for analysis.

### Recombinant *Mtb* antigens

As described previously (Franken *et al*. Protein Expr and Purification 2000), *Mtb* antigens were amplified by PCR from genomic H37RvDNA and cloned by Gateway technology (Invitron, Carlsbad, CA, USA) in a bacterial expression vector containing histidine(His) tag at the N-terminus. Vectors were overexpressed in *Escherichia coli* (*E*.*coli*) BL21 (DE3) and purified. The size and purity of the recombinant proteins were analyzed by gel electrophoresis and western blotting with an anti-His Ab (Invitron) and an anti-*E*.*coli* polyclonal Ab (gift of Statens Serum Institute, Copenhagen, Denmark).

### Data analysis

Antibody levels were presented as OD values. Non-parametric test were used to compare groups. Kruskal-Wallis test with Dunn`s multiple comparisons was used to compare antibody responses among TB patients, HHCs, and CCs at baseline. Mann-Whiney U-test was used to compare antibody responses between patients and contacts at 6 and 12 months. Friedmann test with Dunn`s multiple comparisons was used to compare antibody levels in patients and contacts over time. P values less than 0.05 were considered statistically significant. When three groups were compared simultaneously, p values were adjusted to account for multiple comparisons. GraphicPad Prism version 6.00 for window (GraphPad Software, La Jolle California, USA, http://www.graphpad.com) was used for data analyses.

### Ethical clearance

The study was approved by the Institutional Review Board of Aklilu Lemma Institute of Pathobiology, Addis Ababa University; the National Research Ethics Review Committee of Ethiopia and the Regional Committee for Medical and Health Research Ethics, South-east Norway (Regionale Komite for Medisink og Helsefaglig Forskningsetikk, Sør-Øst), Norway. Written informed consent was obtained from each participant before inclusion into the study.

## Results

**Fig 1:**
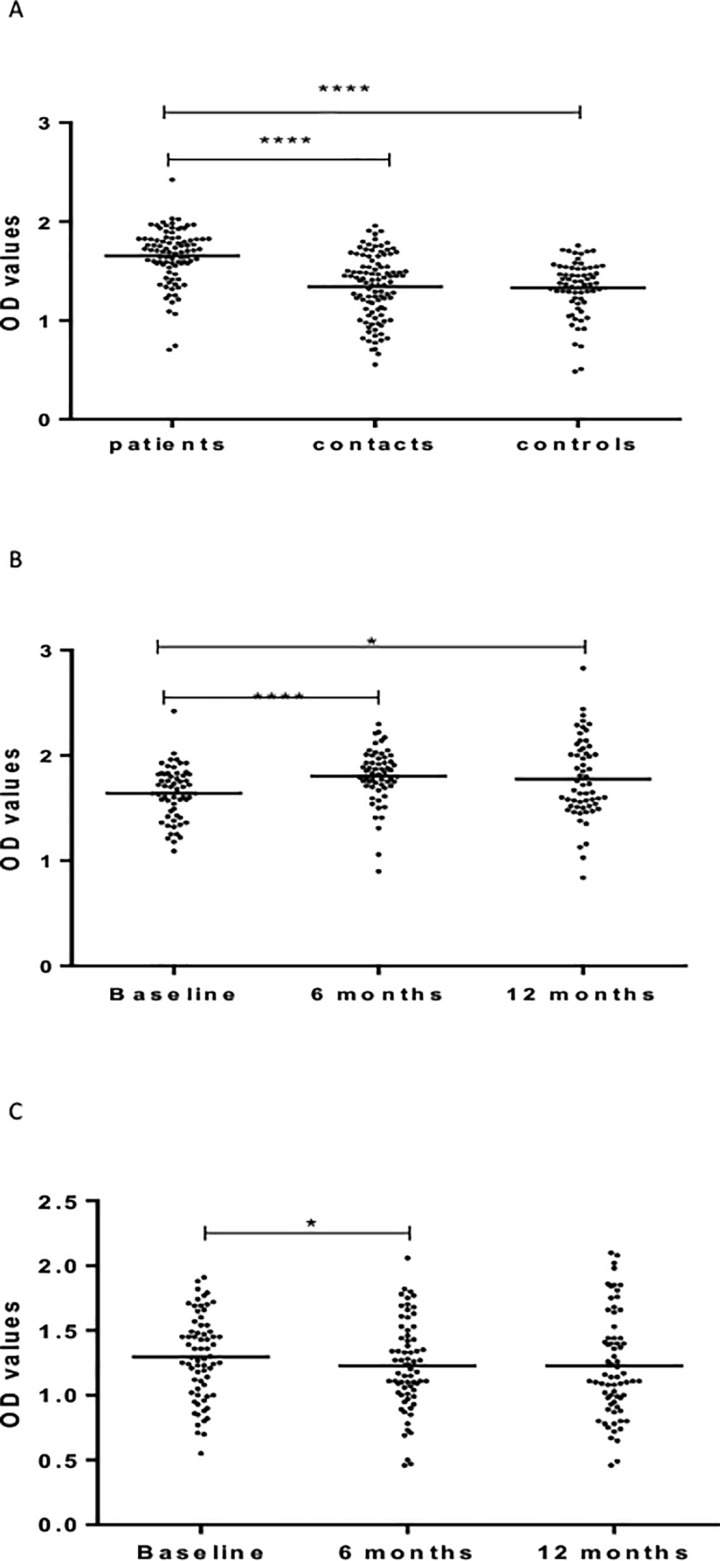
(a-c). IgG responses to LAM at baseline in the three cohorts (Fig a), 6 and 12 months in patients (Fig b) and HHCs (Fig c). Results are individual responses expressed as OD values. The median for each group is shown as horizontal bar. Kruskal-Wallis and Friedman test with Dunn`s multiple comparisons were used to compare antibody responses among groups and over time, respectively. *p<0.05; ****p<0.0001.

Information on socio-demographic characteristics of the study participants and results of QuantiFERON tests has been published elsewhere [35] (Table 1). Fig 1A–1C shows IgG responses against LAM. At baseline, patients had significantly (p < 0.0001) higher levels of IgG against LAM compared to HHCs and CCs. No significant difference was observed between HHCs and CCs (Fig 1A).

**Table 1:** Comparison of QFT results at baseline and 12 months among contacts and patients.

	Patients n(%)		Contacts n(%)	
	Baseline	12 months	Baseline	12 months
**QFT-negative**	7 (21.9*)	6 (18.8*)	**24 (32.4)**	**14 (18.9)**
**QFT-positive**	25 (78.1*)	26 (81.2*)	**50 (67.6)**	**60 (81.1)**
**MacNemar, p-value**	**1.00**		**0.006**	

*One patient with indeterminate result at baseline was excluded from the denominator

Repeated measures of IgG in patients before and after treatment showed a significant (p <0.0001) increase from baseline to 6 months following treatment. Subsequently, there was a non-significant decrease in the level of IgG at 12 months measurements (Fig 1B). In HHCs, the level of IgG against LAM decreased significantly (p < .05) from baseline to 6 months (Fig 1B).

**Fig 2:**
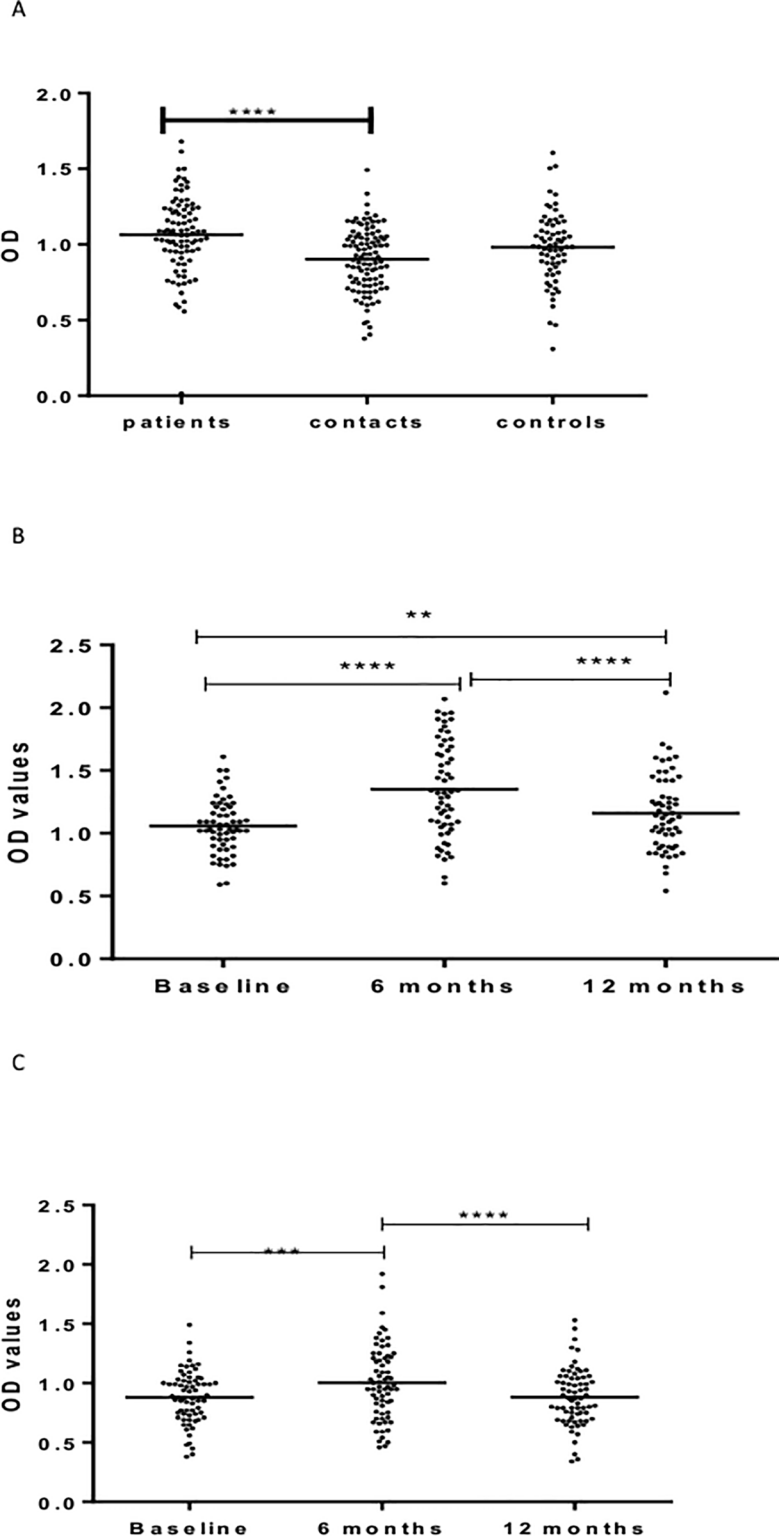
(a-c). IgA responses to LAM at baseline in the three cohorts (Fig a), 6 and 12 months in patients (Fig b) and HHCs (Fig c). Results are individual responses expressed as OD values. The median for each group is shown as horizontal bar. Kruskal-Wallis and Friedman test with Dunn`s multiple comparisons were used to compare antibody responses among groups and over time, respectively. **p<01*p; ***p<0.001; ****p<0.0001.

Fig 2A–2C shows IgA responses to LAM. At baseline, untreated patients had significantly (p <0.0001) higher levels of IgA against LAM compared to HHCs and CCs. There was no significant difference between HHCs and CCs (Fig 2A).

In patients, IgA level increased significantly (p < 0.0001) from baseline to 6 months after treatment but decreased significantly from 6 to 12 months following treatment (Fig 2B).

In HHCs, the level of IgA against LAM increased significantly (p < 0.001) from baseline to 6 month but decreased significantly at 12 month from baseline (p <0.0001) (Fig 2C).

**Fig 3:**
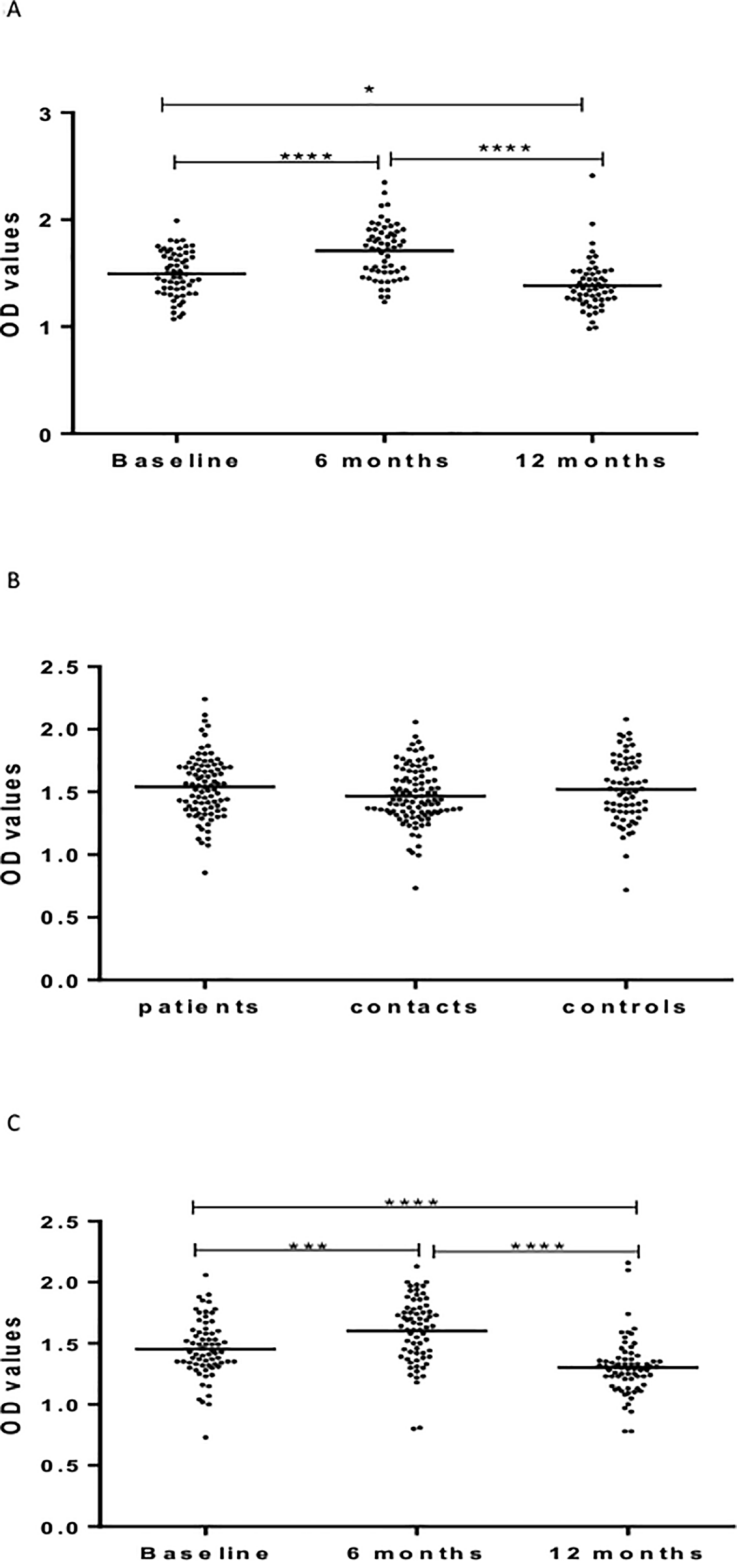
(a-c). IgM responses to LAM at baseline in the three cohorts (Fig a), 6 and 12 months in patients (Fig b) and HHCs (Fig c). Results are individual responses expressed as OD values. The median for each group is shown as horizontal bar. Kruskal-Wallis and Friedman test with Dunn`s multiple comparisons were used to compare antibody responses among groups and over time, respectively. ***p<0.001; ****p<0.0001.

Fig 3A–3C shows levels of IgM against LAM. No significant difference was observed between patients, HHCs, and CCs in the level of IgM against LAM at baseline (Fig 3A).

In patients, the level of IgM against LAM increased significantly (p < 0.0001) from baseline to 6 months but decreased significantly from 6 to 12 months from entry. The level of IgM at 12 months was significantly lower (p < 0.05) compared to the level at baseline (Fig 3B).

In HHCs, the level of IgM against LAM was the highest at 6 months, followed at baseline and the lowest at 12 month from entry (Fig 3C).

**Fig 4:**
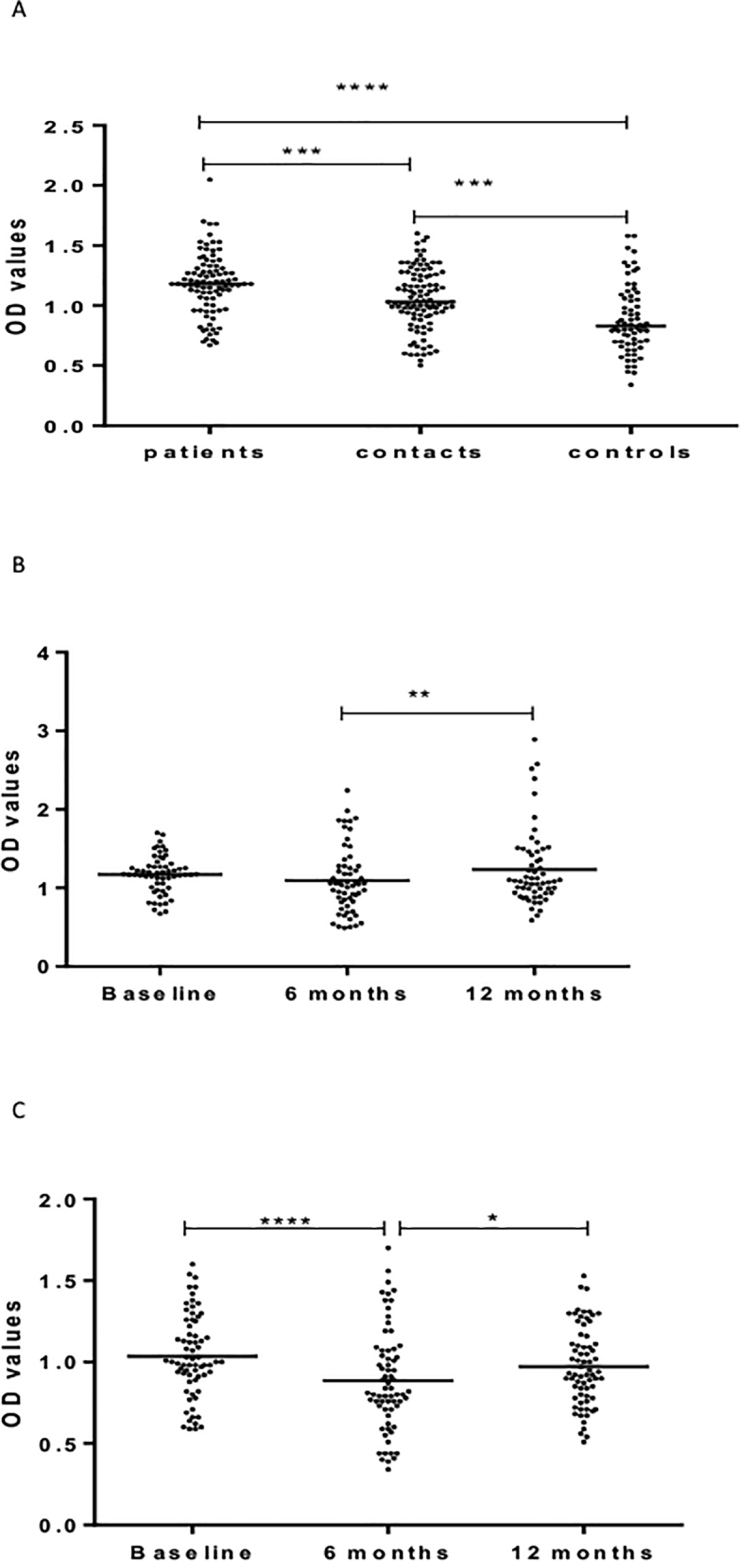
(a-c). IgG responses Rv2031 at baseline (Fig a), 6 and 12 months in patients (Fig b) and HHCs (Fig c). Results are individual responses expressed as OD values. The median for each group is shown as horizontal bar. Kruskal-Wallis and Friedman test with Dunn`s multiple comparisons were used to compare antibody responses among groups and over time, respectively.*p0.05; **p<0.01; ***p<0.001; ****p<0.0001.

Fig 4A–4C shows results of IgG response to Rv2031. At baseline, the level of IgG was significantly different between the three cohorts, being the highest in untreated patients, followed by HHCs, and the lowest in CCs (Fig 4A).

Kinetic measurements in patients showed that IgG levels increased significantly (p<0.01) 12 months following treatment compared to baseline or 6 months after treatment (Fig 4B).

However, in HHCs, the level of IgG against Rv2031 decreased significantly (p< 0.0001) at 6 months but increased significantly (p < 0.05) at 12 months from entry (Fig 4C).

**Fig 5:**
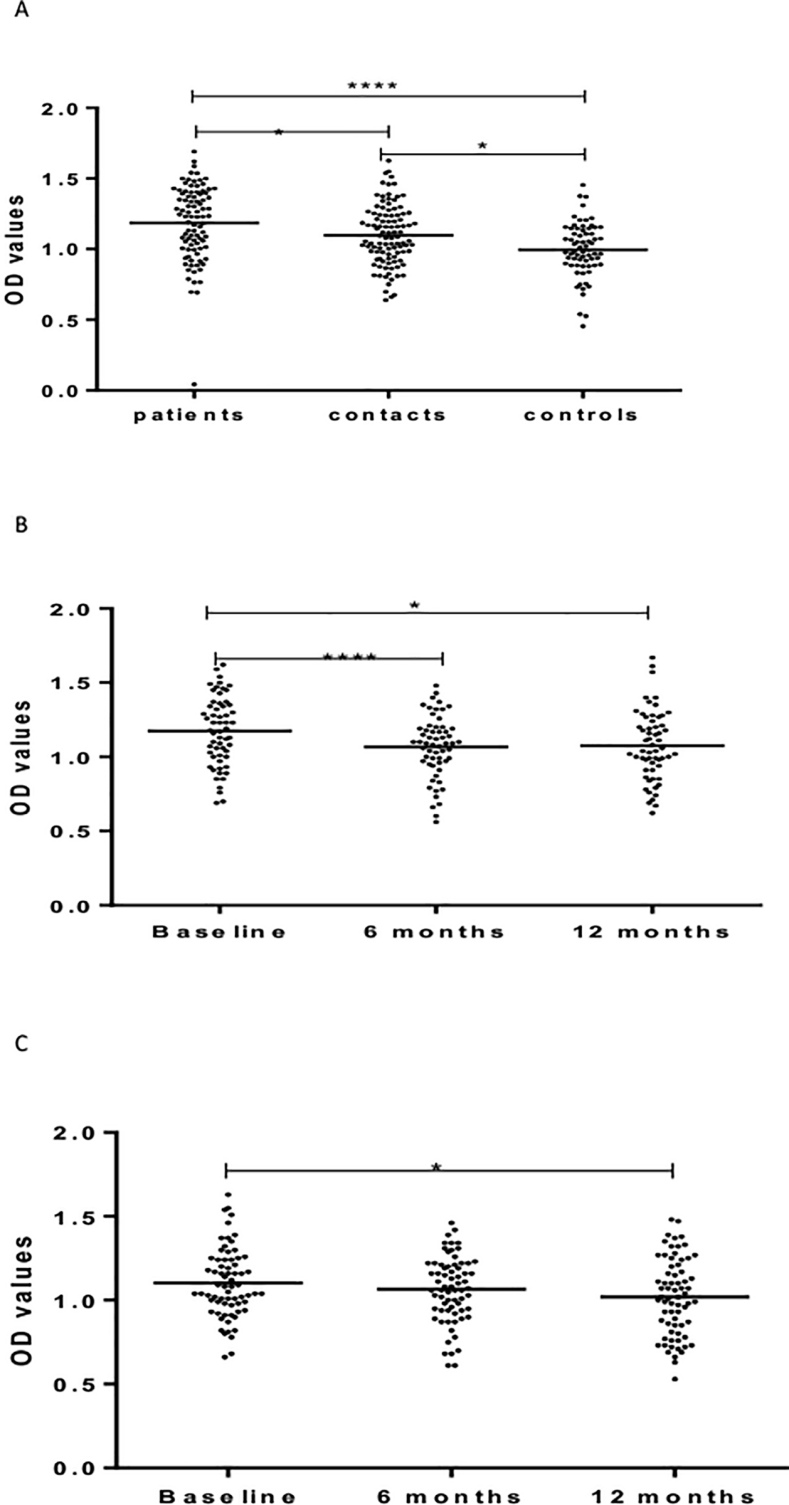
(a-c). IgA responses to Rv2031 at baseline (Fig a), 6 and 12 months in patients (Fig b) and HHCs (Fig c). Results are individual responses expressed as OD values. The median for each group is shown as horizontal bar. Kruskal-Wallis and Friedman test with Dunn`s multiple comparisons were used to compare antibody responses among groups and over time, respectively. *p<0.05; ****p<0.0001.

Fig 5A–5C shows IgA responses to Rv2031. At baseline, the level of IgA against Rv2031 was significantly (p < 0.0001) higher in patients compared to CCs, and HHCs (p < 0.05). HHCs had significantly higher (p < 0.05) higher levels of IgA than CCs (Fig 5A).

The level of IgA decreased significantly (p< 0.0001) from baseline to 6 months and remained low following treatment in patients (Fig 5B).

In HHCs, the levels of IgA was significantly (p < 0.05) higher at baseline compared to 6 and 12 months from entry but there was no difference between 6 and 12 months (Fig 5C).

**Fig 6:**
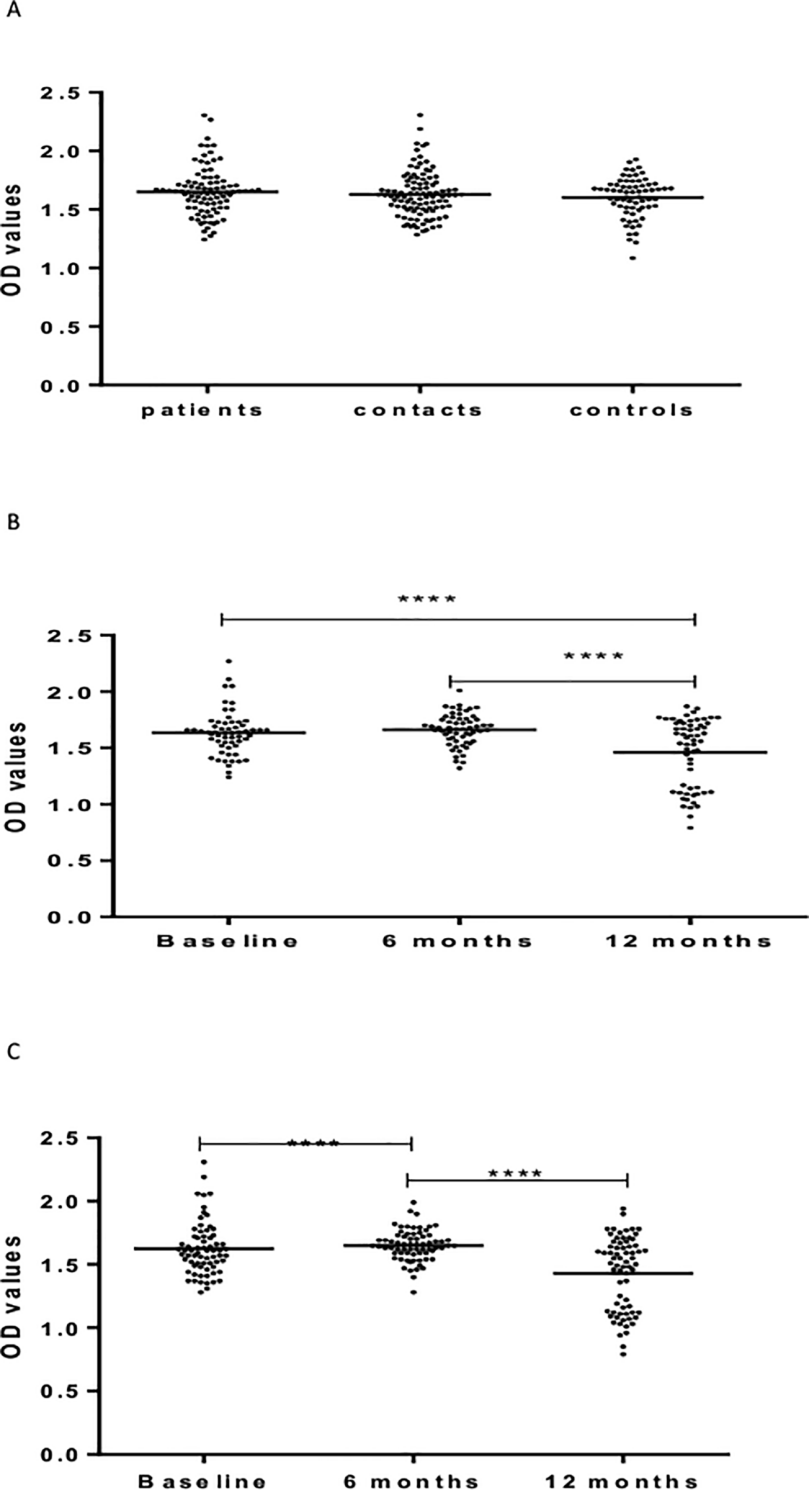
(a-c). IgM responses to Rv2031 at baseline (Fig a), 6 and 12 months in patients (Fig b) and HHCs (Fig c). Results are individual responses expressed as OD values. The median for each group is shown as horizontal bar. Kruskal-Wallis and Friedman test with Dunn`s multiple comparisons were used to compare antibody responses among groups and over time, respectively. ****p<0.0001.

Fig 6A–6C shows IgM responses to Rv2031. There was no difference between patients, HHCs and CCs in the level of IgM at baseline (Fig 6A). In patients, there was no significant difference between baseline and 6 months following treatment. However, the level of IgM decreased significantly (p< 0.0001) 12 months following treatment (Fig 6B). In HHCs, the level of IgM against Rv2031 increased from baseline to 6 months (p<0.0001) but decreased again at 12 months from baseline (p<0.0001) (Fig 6C).

**Fig 7:**
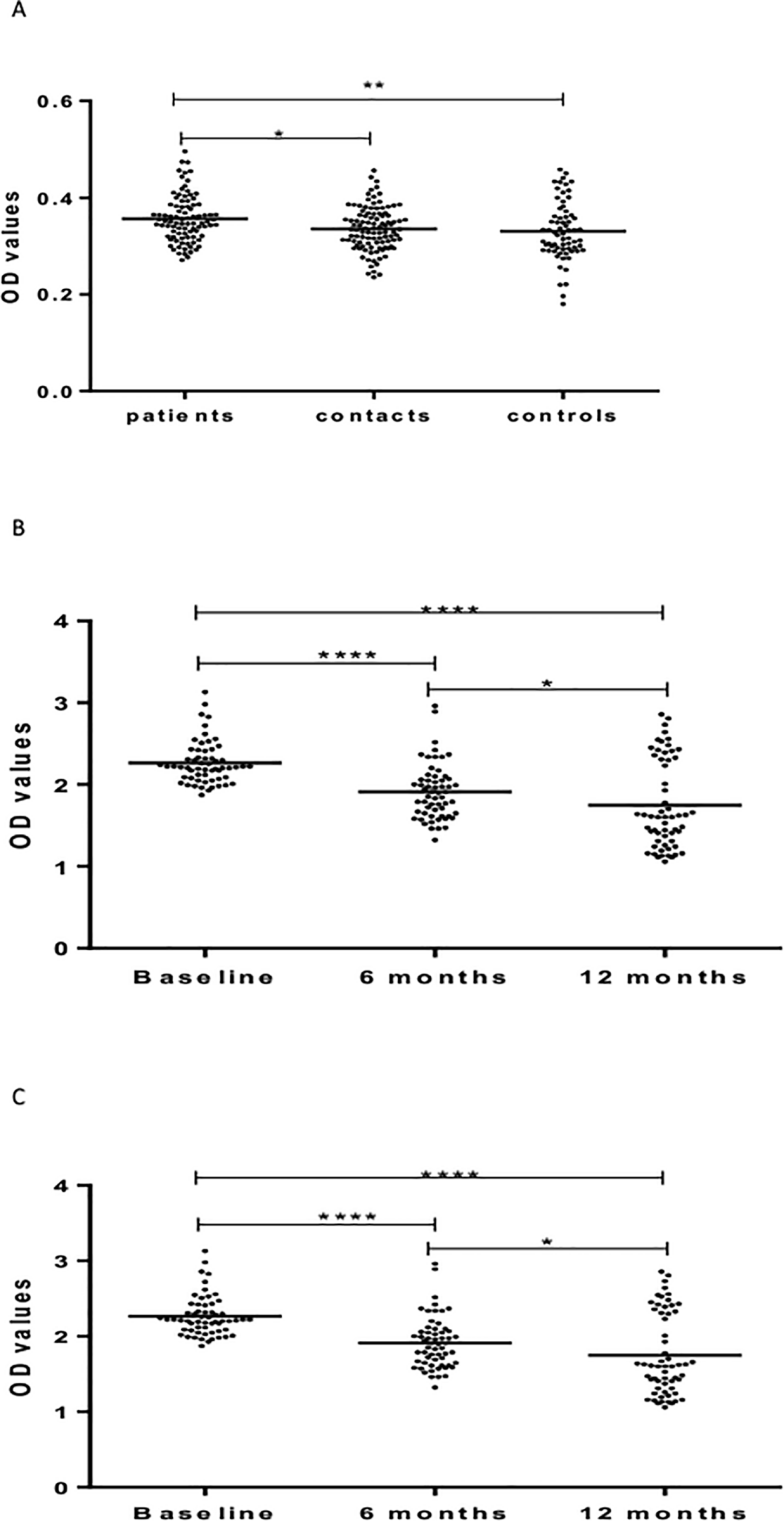
(a-c). IgG responses to HBHA at baseline (Fig a), 6 and 12 months in patients (Fig b) and HHCs (Fig c). Results are individual responses expressed as OD values. The median for each group is shown as horizontal bar. Kruskal-Wallis and Friedman test with Dunn`s multiple comparisons were used to compare antibody responses among groups and over time, respectively. ****p<0.0001.

Fig 7A–7C shows IgG responses to HBHA. At baseline, patients had significantly higher levels of IgG against HBHA than HHCs (p < 0.05) and CCs (p < 0.01) (Fig 7A). However, the levels of IgG against HBHA decreased significantly and progressively 6 months (p < 0.0001) and 12 months (p< 0.05) following treatment (Fig 7B). There was a similar significant and progressive decline over a period of 12 months in the level of IgG against HBHA in HHCFs (Fig 7C).

**Fig 8:**
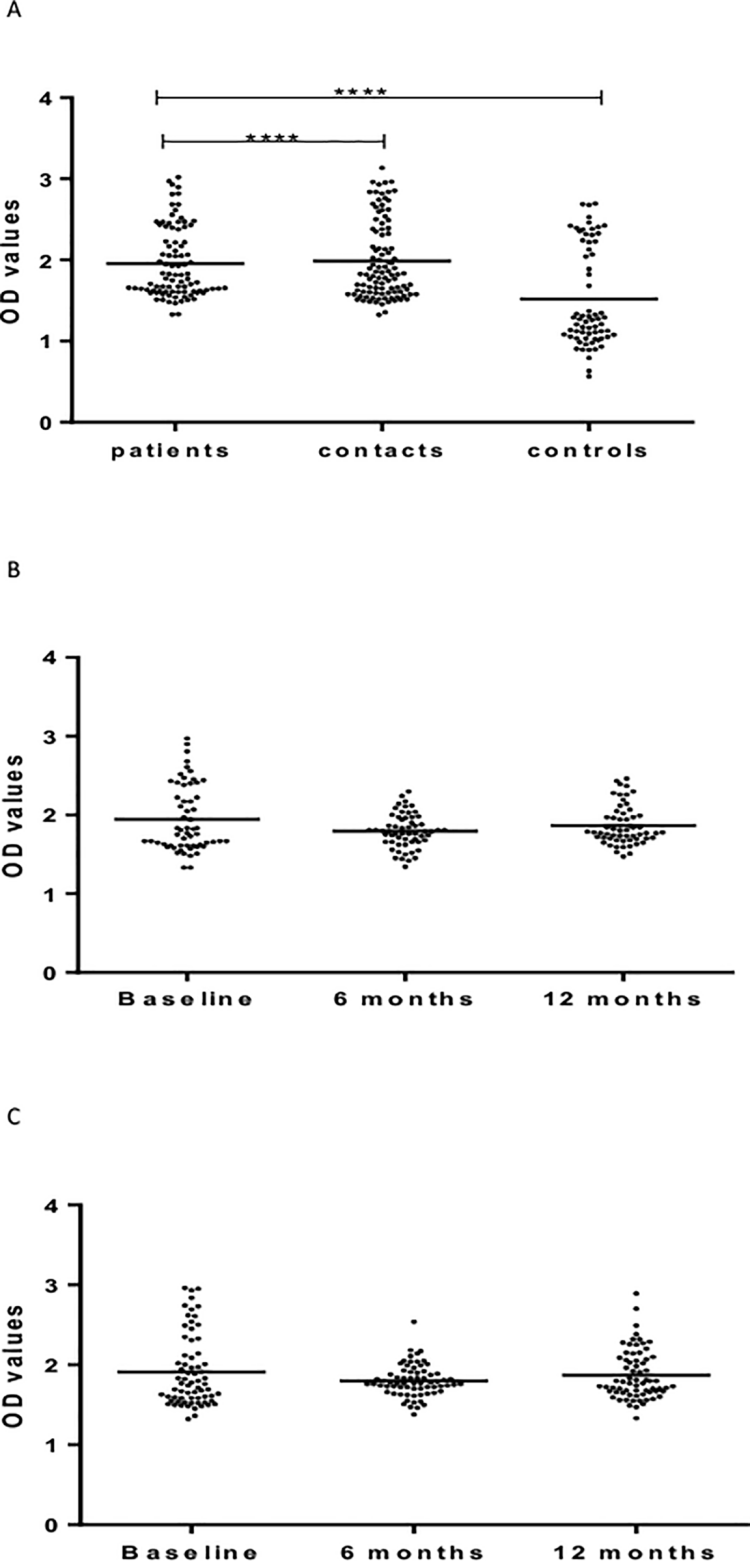
(a-c). IgM responses to HBHA at baseline (Fig a), 6 and 12 months in patients (Fig b) and HHCs (Fig c). Results are individual responses expressed as OD values. The median for each group is shown as horizontal bar. Kruskal-Wallis and Friedman test with Dunn`s multiple comparisons were used to compare antibody responses among groups and over time, respectively. ****p<0.0001.

Fig 8A–8C shows the level of IgM against HBHA. At baseline, the level of IgM against HBHA was significantly higher in patients and HHCs (p< 0.0001) compared to CCs but there was no significant difference between patients and HHCs (Fig 8A). However, repeated measures in both patients and HHCs did not show any significant difference (Fig 8B & 8C). Results of IgA responses to HBHA have been published elsewhere [2]

## Discussion

In the present study, we compared IgA, IgG, and IgM responses to *Mtb* antigens, LAM, Rv2031, and HBHA in cohorts of PTBP, HHCs, and CCs in an endemic setting. The results show that the levels of IgA and IgG against Rv2031 were significantly higher in untreated PTB patients, followed by HHCs and the lowest in CCs. This may imply that there is a strong correlation between the levels of IgA and IgG with bacillary load. Earlier, in a study carried out in a pastoral community, our group reported that IgA against ESAT-6/CFP-10 and Rv2031 discriminated between pulmonary TB patients, healthy-*Mtb-*infected and non-infected individuals [27]. These results are in agreement with other studies that showed antibody levels against *Mtb* components are markers for bacterial load [38], which are in turn associated with risk of disease [39–41]. Results of other studies [42–44] also suggest that IgA against Rv2031 could discriminate between clinical TB patients and healthy controls. In addition, a study carried out by Kunnath-Velayudhan *et al* [39] in which TB suspects were stratified into groups of absent, low and high, based on results of sputum-smear microscopy and bacterial load, have shown that antibody levels correlated with *bacillary* load. Recently, Baumann *et al* [45] have shown that IgA levels against L-alanine dehydrogenase AlaDH (Rv2780), nitrate/nitrite response transcriptional regulator NarL (Rv0844c), 19-kDa lipoprotein antigen precursor LpqH (Rv3763), periplasmic phosphate-binding lipoprotein pstS3 (Rv0928), and MPT83 (Rv2873) were consistently elevated in a small control sub-group, and suggested that IgA, together with IgG could have a prognostic potential. The above reports have been further augmented by a recent study [46], which has documented that IgA and IgG against selected mycobacterial antigens provide promising diagnostic signatures for active TB. Put together, results of all the above studies from different geographic communities consistently suggest that *Mtb* antigen specific IgA and IgG could be used to develop an accurate and simple ELISA test for the diagnosis of TB.

Second, results of this study show that there are significant variations in the levels of antibody isotypes against the three antigens in PTB patients (before and after treatment) and contacts over time. For instance, while the levels of IgG and IgA against Rv2031 (heat shock protein) were significantly different between PTB patients, HHCs and CCs (Figs 4A & 5A), no significant difference was observed in the levels of IgM against LAM between untreated patients, HHCs, and CCs (Fig 3A). Moreover, the level of IgM against HBHA was similar in untreated patients and HHCs but was significantly lower in CCs (Fig 8A). Earlier, our group [9] has shown that healthy community controls had a significantly higher (p<0.0001) level of IgA against HBHA compared to untreated patients, implying that IgA against HBHA could be a marker for protective immunity against TB. Therefore, results of the present study suggest that not all antibody responses are markers of clinical TB [29] or carry risk of disease progression as suggested earlier [39–41].

Such differences are primarily related to structural, antigenic and functional differences in constant region of heavy chain of antibody isotypes. For instance, the fact that the levels of IgA and IgG against Rv2031 (heat shock protein) increased with antigen load could be due to affinity maturation of activated B cells/isotype switching and production of high affinity IgA and IgG. IgG is the most abundant antibody isotype in plasma because it is produced by plasma cells derived from B cells that have undergone class switching [47]. However, the fact that the level of IgM against LAM did not show any significant difference between PTB patients, HHCs, and CCs (with different bacillary load) could be because LAM being a mycobacterial glycolipid, may not induce affinity maturation of B cells and class switching (which requires T cell help) in the same manner as protein antigens.

There was also a significant variation in antibody responses against these antigens in patients before and following chemotherapy. Some antibody isotypes (e.g. IgG against HBHA, IgA and IgM against Rv2031) decreased significantly, while others (IgA against LAM, and IgM against LAM) increased 6 months following chemotherapy, while still others (IgG against Rv2031, IgM against HBHA) did not show any significant change 6 months after treatment.

There are two plausible explanations for the increase in antibody responses following chemotherapy. The fact that some antibody isotypes (IgG and IgA against LAM) increased following treatment and remained high 12 months following treatment may suggest reinfection in this endemic area, whereas the transient increase in antibody responses (IgM against LAM and Rv2031) might be explained by disintegration of the bacteria and associated release of antigens, which may result in an increase in antibody isotype responses. Earlier, Mattos *et al*. [48] have reported that the level of antibody responses against some antigens (16-kDa antigen) increase temporarily following chemotherapy and attributed this transient increase to disintegration of the bacilli and release of cytosolic antigens. In a study in *Schistosoma haematobium* infection in Zimbabwe, it was shown that chemotherapy has an immunizing effect, in addition to a transient reduction in the level of infection [49]. However, the decline in the levels of some antibody responses (IgA and IgG against RV2031 and HBHA) following chemotherapy can be explained by the removal of the pathogen and decrease in bacillary load. In favor of our view, several studies have reported that the level of IgG against Rv2031 decreased significantly following chemotherapy and a decrease in bacterial load [50–52]. Imaz *et al* [52] have reported that the level of antibodies (IgG) to different mycobacterial antigens decreased significantly after anti-tuberculous treatment compared to baseline. Baumann *et al* [46] have also reported that patients with elevated levels of antibody isotypes before treatment respond to treatment slowly.

Variations in antibody responses are not limited to pulmonary TB patients. However, there were significant variations in antibody responses in HHCs over time. Some antibody levels decreased from baseline to 6 months and remained the same (IgG against LAM); some increased from baseline to 6 months and returned to the original level at 12 months (IgA, IgM against LAM; IgA against Rv2031); and some decreased significantly and progressively over 12 months period (IgG against HBHA).

It is difficult to provide definitive explanations but one possible explanation for the increase over time of some antibody isotypes could be due to disease progression, super-infection or infection by environmental mycobacteria. Our results are supported by results from different studies [53–55]. In a study carried out in Spain [54], it was reported that antibody response to the 16-kDa antigen was non-specific for the control of non-TB pneumonia population. Similarly, Raja *et al* [55] have reported that 31% of individuals with non-TB lung diseases have antibodies to the 16-kDa antigen.

There are reports that show cross-reactive antibodies generated by exposure to environmental mycobacteria or non-pathogenic enteric or pulmonary bacteria [56, 57]. It has also been shown that the development of low level humoral immune responses to *Mycobacterium avium* sonic extracts and mycobacterial LAM in children correlates with increasing age, and by 18 years of age many individuals were sero-reactive to mycobacterial antigens [58].

### Conclusion

Results of the current study involving a large sample of PTBP, their HHCs and CCs show that the levels of IgA and IgG against Rv2031 were significantly higher in PTBP compared to HHCs and CCs. The levels of these antibody isotypes were also significantly higher in HHCs compared to CCs. The results suggest that IgA and IgG against Rv2031 discriminate between clinical TB patients, *Mtb-*infected and non-infected individuals, implying the potential for the diagnosis of tuberculosis. The fact the levels of IgA, IgG, and IgM varied significantly for the different antigens and cohorts may suggest that not all antibody responses are markers of clinical TB as suggested earlier.
